# Spatial and climatic variables independently drive elevational gradients in ant species richness in the Eastern Himalaya

**DOI:** 10.1371/journal.pone.0227628

**Published:** 2020-01-15

**Authors:** Aniruddha Marathe, Dharma Rajan Priyadarsanan, Jagdish Krishnaswamy, Kartik Shanker

**Affiliations:** 1 Ashoka Trust for Research in Ecology and the Environment (ATREE), Srirampura, Bangalore, India; 2 Manipal University, Manipal, India; 3 Centre for Ecological Sciences, Indian Institute of Science, Bangalore, India; University of Delhi, INDIA

## Abstract

Elevational gradients are considered important for understanding causes behind gradients in species richness due to the large variation in climate and habitat within a small spatial extent. Geometric constraints are thought to interact with environmental variables and influence elevational patterns in species richness. However, the geographic setting of most mountain ranges, particularly continuity with low elevation areas may reduce the effect of geometric constraints at lower elevations. In the present study, we test the effects of climatic gradients and continuity with the low elevation plains of the eastern Himalayan mountain range on patterns of species richness. We studied species richness of ants (Hymenoptera: Formicidae) on an elevational gradient between 600m and 2400m in the Eastern Himalaya–part of Himalaya biodiversity hotspot. Ants were sampled in nine elevational bands of 200m with four transects in each band using pitfall and Winkler traps. We used regression models to identify the most important environmental variables that predict species richness and used constrained null models to test the effects of contiguity between the mountain range and plains. We find a monotonic decline in species richness of ants with elevation. Temperature was a more important predictor of species richness than habitat complexity. Geometric constraints model weighted by temperature with a soft lower boundary and hard upper boundary best explained the species richness pattern. This suggests that a combination of climate and geometric constraints drive the elevational species richness patterns of ants.

## Introduction

Elevational gradients are considered as ideal natural laboratories for understanding processes that limit and maintain ecological communities because of the large variation in environmental conditions with concurrent changes in biological communities [1]. Research on ecological communities on elevational gradients has laid the foundations of the niche concept [2], gradient analysis [3–5] and beta diversity [4]. Studies and reviews across multiple scales for a large number of taxa show that the pattern in species diversity across elevations is not uniform and may decrease, peak at mid-elevations, or in rare cases, increase with elevation [6–12]. The hypotheses considered for explaining the patterns are broadly climatic such as 'elevational Rapoport's rule' [13,14], energy limitation hypothesis [15,16], and temperature and moisture availability [17–19]; ecological such as the 'ecotone effect' [20]; evolutionary such as the isolation of mountain tops leading to higher extinction rates [21], in situ speciation [22], and niche conservatism [23]; and geographical, namely geometric constraints or mid-domain effect [24–25]. A large number of possible explanatory mechanisms together with different elevational diversity patterns among mountains and taxa suggest that the effects of multiple mechanisms are influencing the gradients rather than a single unifying explanation.

A number of studies on ants from the tropics report decrease in species richness with elevation [26–30], while some others report mid-elevation peaks [31–33]. Temperature [34,35], area of elevation bands along with geometric constraints [36] and climatic stability [37] have been found to be important in determining ant species richness across elevations. A global analysis of elevational patterns in ant diversity indicates that the patterns are driven by a complex interplay of multiple factors, particularly temperature and precipitation along with geometric constraints [12]. Such factors very likely limit communities at coarse grain sizes limiting the species pools, while conditions of habitat availability, complexity, and micro-climate may further limit species richness of local communities. Some studies have tested effects of habitat and elevation simultaneously [26], but the hierarchical nature of these effects has not been tested on ant communities across elevation gradients. In this study, we test the hierarchical effects of climate and habitat on ant communities using mixed effects regression models. We then use the relevant variables in a geometric constraints model with varying assumptions of boundaries to understand how geometric constraints affect patterns in species richness.

Most studies test geometric constraints on elevational gradients using random unconstrained models with hard boundaries and the few that combine climatic predictor variables with geometric constraints also assume that all species are confined within the observed spatial extent [12,38] (but see Rana et. al [39] for a conceptual model). However, elevational extents present in most empirical data are likely to be subsets of actual geographical extents of species. Therefore, the assumption of hard boundaries is not entirely valid and ‘niche truncation model’ (sensu [40]), which includes opportunities for species crossing the domain boundaries, should be the appropriate null model to test the patterns. Hence, in this study, we test between geometric constraints models that differ in assumption of hard boundaries and explanatory variables.

We carried out this study on an elevation gradient that contains tropical evergreen and subtropical forests in the Eastern Himalaya which is a region of global importance for biodiversity conservation [41]. Ants were sampled from multiple locations across a single mountain slope to understand the processes that drive elevational patterns in species richness. We used (a) a conventional analytical approach (regression models) to examine the effect of a suite of environmental and habitat variables, and (b) a simulation and modelling approach using constrained null models to integrate geometric constraints and environmental variables to explain the elevational gradient in species richness.

## Materials and methods

### Ethics statement

This work involved field research and the appropriate permissions were provided by the Arunachal Pradesh Forest department (Ref: CWL/G/13 (17)/06-07/12-14; dated 6th Jan 2010). AM consulted the village elders in the field area before starting the research.

### Study site

**Fig 1:**
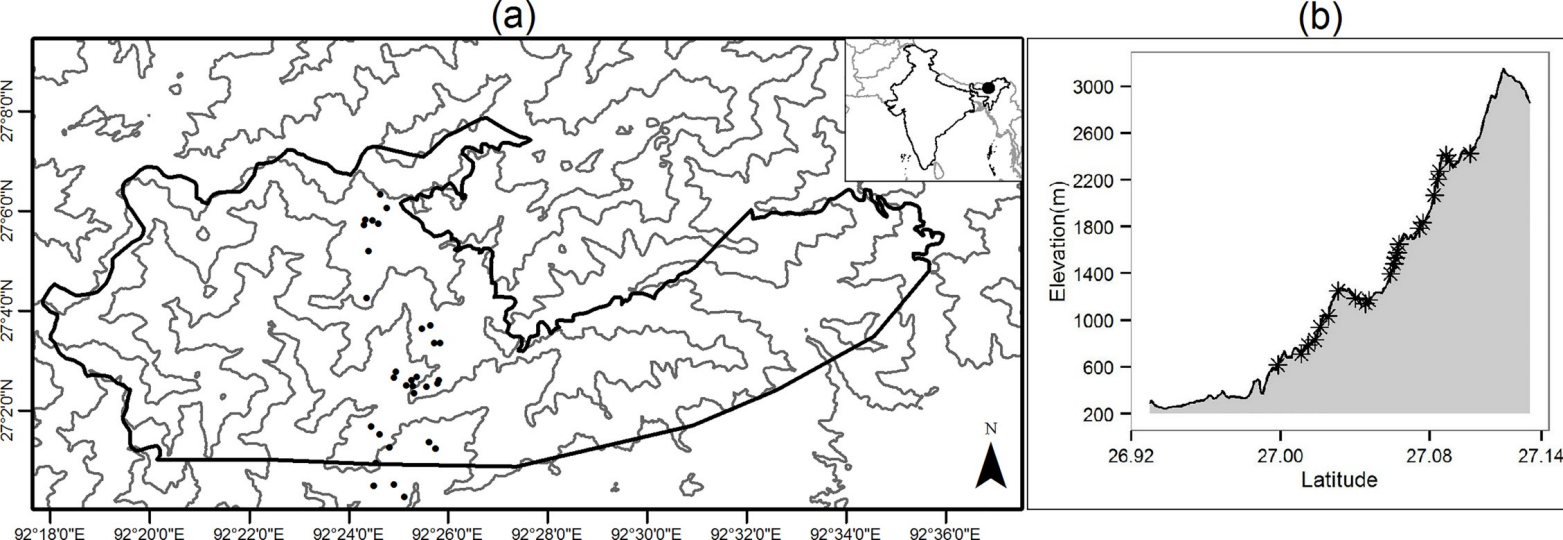
Map of study area. (a) Sampling locations within Eaglenest wildlife sanctuary (EWS) and (b) Elevation profile of the sampling locations.

This study was conducted in the Eaglenest Wildlife Sanctuary (EWS) located in the state of Arunachal Pradesh, India. EWS covers a wide elevation range from 500m to 3250m (Fig 1). The temperature at the lowest elevations is as high as 30°C during summer (April—May) but drops sharply with elevation to about 15°C at 2400m during the same period. Winter temperatures range from 15°C to 3°C across the same elevation range according to 'WorldClim data' [42]. Vegetation in the sanctuary is broadly tropical evergreen, sub-tropical and temperate broadleaved [43]. Rhododendron stands and small patches of coniferous forest are present near the highest elevations. However, site-specific vegetation data does not exist. Most of the sanctuary is free of any recent disturbance except some areas at the lowest elevations.

### Sampling design

All sampling locations were on the south facing slope of the EWS ridge. We classified the elevation gradient into nine broad classes of elevations or elevation bands between 600m and 2400m at 200m intervals, and sampled each of these bands during the summer (April—May) of 2013. Within each elevation band, four replicate transects of 100m length were established separated by 300m to 1000m. Sufficient care was taken so that the difference in actual elevation between transects within an elevation band was less than 50m. All transects were sampled with 10 pitfall traps each. Plastic jars with 10 cm diameter mouth were used as pitfall traps. Each trap had a mixture of 90% alcohol as fixative. A few drops of glycerol were added to prevent evaporation of alcohol. The jars were buried in the ground such that the lip of the jar was in level with or slightly below the ground. Ten such pitfall traps (10 m apart) were placed on each transect. Pitfall traps were open for 48 hours. Two of the four transects in each were sampled with 20 Winklers each while the other two were sampled with 10 Winklers, made as per standard specifications [44] Sampling among replicates within elevation s was uneven due to logistic constraints. We used sample based rarefied richness for regression analysis to account for difference in sampling.

### Analysis

#### Regression analysis

Since transects are nested within elevation bands, we used hierarchical regression analysis using generalized linear mixed models with Poisson errors to identify the variables explaining the most variation in species richness. The mixed effects regression models had different predictors at elevation bands and transects.

We used rarefied species richness at each transect as a response variable for regression analysis. Pitfall traps and Winkler collections differ in rates of species accumulation and collect nested assemblages [45]. Therefore, using these different methods as replicates for estimating species richness is not meaningful. Hence, for regression analysis, we used data only from Winklers and rarefied the species richness to a common sample size of ten Winkler samples at each transect. We used the package 'INEXT v2.0' [46] for obtaining rarefied species richness. We also analysed the observed species richness at each transect, and rarefied and interpolated species richness at elevation bands using generalized linear models with Poisson errors (Table 1–4 in S1 Text).

We checked for spatial auto-correlation in species richness using Moran's I and by checking autocorrelation in the residuals from the regression model of species richness with elevation as the covariate. If the dependence of species richness on a possible explanatory variable across the elevational gradient is sufficient to account for most of the spatial dependence in species richness, then the regression residuals should not have any spatial autocorrelation [47].

We estimated volume of leaf litter, and complexity of understory vegetation to represent local habitat complexity. We also measured soil temperature at the time of sampling, which we used only for generalized linear models. For the mixed effects regression, we used climatic variables obtained from ‘WorldClim’ at 30 arc second resolution as predictors at the level of elevation bands. Details of methods for estimating the predictor variables are presented in supplementary text.

We compared regression models that included effect of either climatic variables or habitat complexity and one model that included both variables. The models were compared using AICc. We used 'R v3.4' [48] to carry out all analyses. We used package 'spdep v0.5–88' [49] for spatial analysis and package 'lme4 v1.1' [50] for regression analysis.

### Geometric constraints models

For testing the effect of geometric constraints on species richness, we used simulation models that randomly distribute species across elevations. In the absence of any additional mechanism, the model will predict a unimodal pattern in species richness as long as species ranges are contiguous and entirely confined within the observed part of the gradient [24].

The hypotheses for range contiguity, effect of boundaries, and presence of limiting environmental variables, can be tested by modifying the rules with which species are distributed [51]. We decided to interpolate species occurrences between lowest and highest points after comparing the number of discontinuous occurrences in the observed data and null model (S1 Text). We randomized the elevational extents of each species by randomly selecting mid-points from all possible values based on the nature of boundaries and geometric constraints. We used the observed range size frequency distribution so that the number of elevation bands occupied by a species was the same as the observed data but range locations were randomized [38]. When the model is not constrained by any variable, every possible midpoint has an equal probability of selection. In the case of a constraining explanatory variable, the probabilities of selecting range mid points are weighted by the respective values of the variable. Other studies have used species with small ranges, which are not affected by geometric constraints, to estimate effects of climatic predictors. [12,38]. We did not use this method as species with small ranges (up to three elevation s) constitute more than 50% of the species pool.

In addition to constraining the null model, we also changed the limits of geometric constraints. Models with hard boundaries assume that the sampled domain completely covers the range limits of all species, so no species can cross the domain boundaries. In a hard boundary model, only geometrically possible range locations are available for sampling. On the other hand, in the niche truncation model (sensu [52]), all possible range locations are available for sampling so that some species may cross the domain boundaries. Range locations of such species are then adjusted to the nearest geometrically feasible value. Here, the assumption is that the observed extents of species are truncated subsets of actual distributions that extend beyond the domain boundary [39]. This model is similar to the range spread model where chances for species occurring at domain boundaries are much higher compared to hard boundary models [40,52].

Given that the spatial scale of the study area is relatively small compared to the entire elevational gradient in eastern Himalaya, elevational extents sampled during this study are likely to be smaller than the entire elevational range of the species, as at least some species may extend beyond the 600m to 2400m bounds of this study. Earlier studies have dealt with problems of truncated elevational or spatial extents by augmenting data from literature [39]. However, since there is little distributional data available for ants, we considered the chance of occurring below or above the sampled elevational extent as uniform for all species. To account for this, we considered the domain as 200m to 2800m for all geometric constraints models. However, the proportion of elevational extent occupied by each species within the sampled elevational range is the same as in the data, and we compare the predicted richness within the sampled elevational range only.

We used the variants of the geometric constraints model that differed in three parameters—range contiguity, nature of boundaries and climatic gradients (Table 1; Fig 1 in S1 Text). Models where ranges are not contiguous correspond to ‘Range Scatter’ models, while those with contiguous ranges correspond to ‘Range Cohesion’ models (*sensu* [53]).

**Table 1:** Description of geometric constraints models used based on the nature of hard boundaries and the domain. All models in the table are range cohesion models while model 1 is the only range scatter model.

	Nature of boundaries		
	Both boundaries soft	Both boundaries hard	Lowe boundary soft while upper hard
Elevational domain extended on low as well as high ends	Model 3	Model 2	Model 4
Elevational domain extended on only at low elevation			Model 5

We calculated residual deviations of all candidate models and considered model1 as the biologically relevant null model to calculate R^2^ values. We also report R^2^ values by using null deviance from the mean of species richness in supplementary text (S1 Text). We used package 'rangemodelR v1.0.4' (https://CRAN.R-project.org/package=rangemodelR) in 'R v3.4' [48] for running geometric constraints models.

## Results

**Fig 2:**
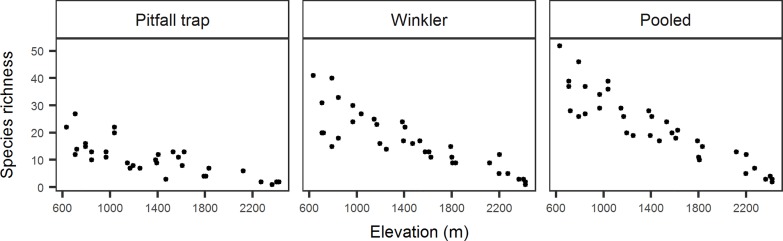
Observed species richness of ants across elevation. (a) pitfall trap, (b) Winklers, (c) pooled.

**Fig 3:**
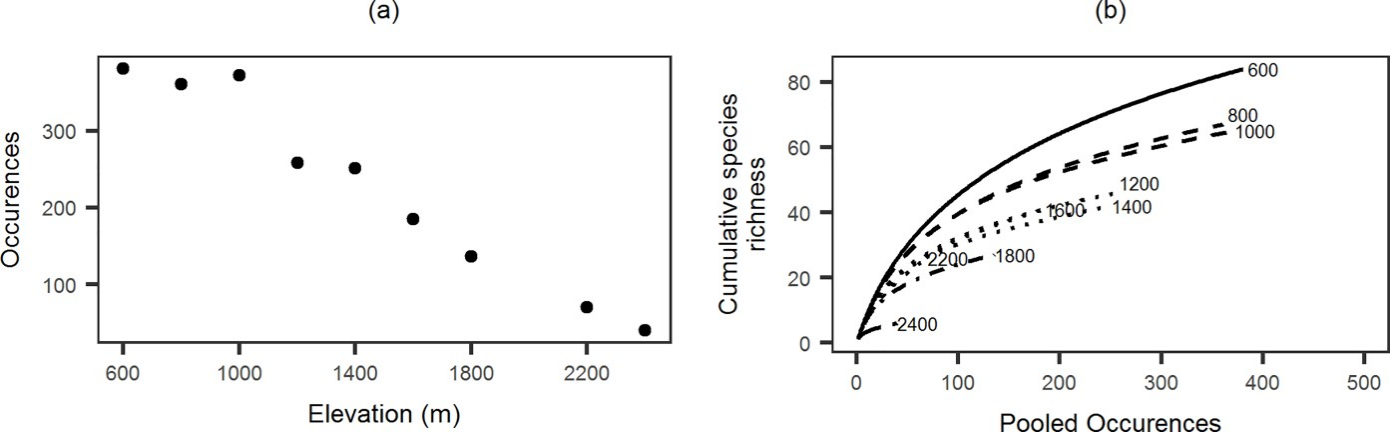
Rarefaction for ant communities at each elevation band. (a) Total number of occurrences recorded at each elevation, (b) rarefaction curves with cumulative species richness of ants and cumulative number of occurrences for each elevation.

**Fig 4:**
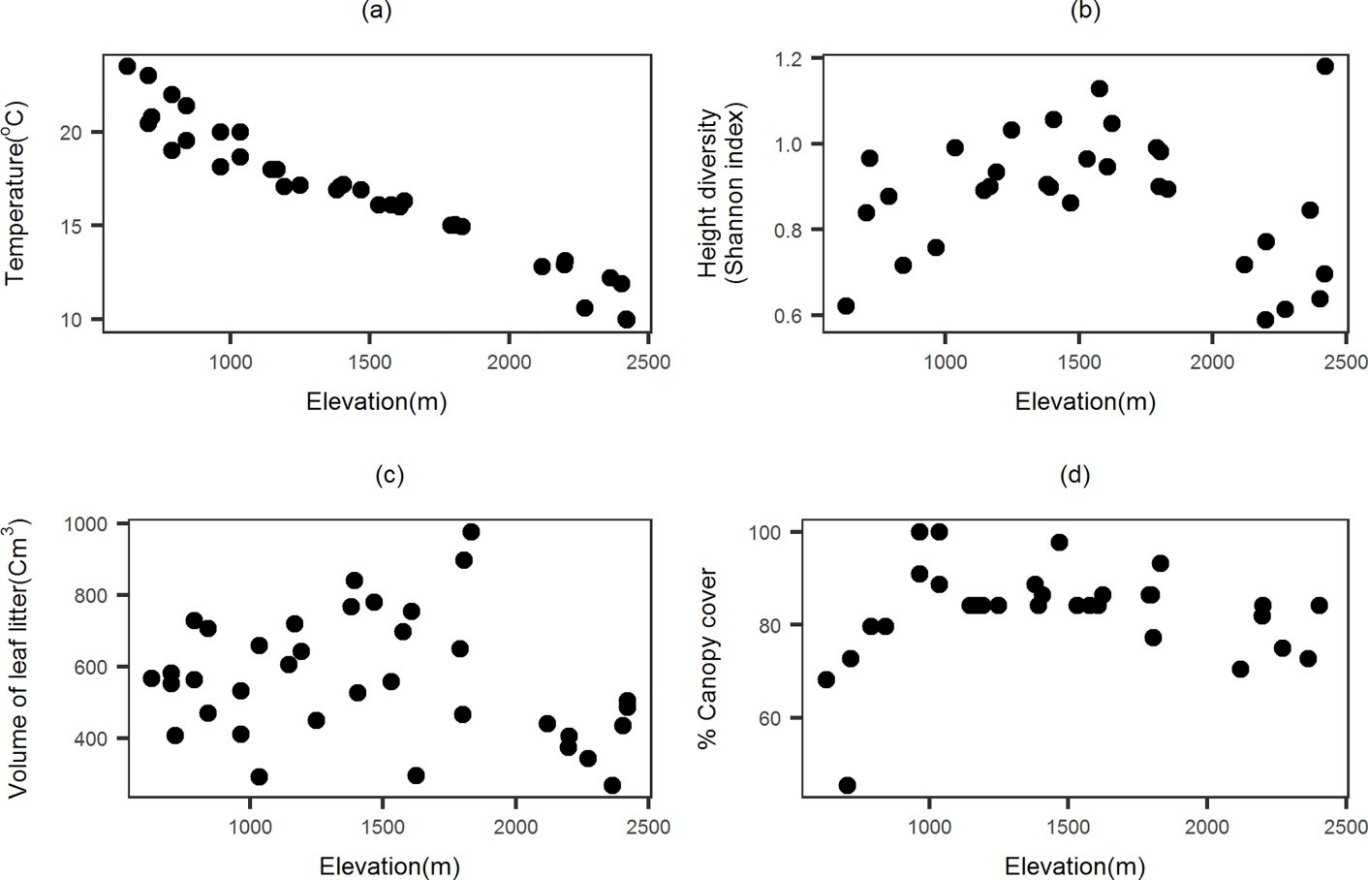
Pattern of predictor variables across elevation. (a) temperature, (b) canopy cover, (c) volume of leaf litter, and (d) vegetation complexity.

We collected 10,560 individuals belonging to 157 species and 51 genera. Observed species richness decreased with an increase in elevation, without any indication of a peak at mid elevations. The pattern was consistent for data specific to each of the two collection methods as well as for the pooled data (Fig 2A–2C). The total number of species occurrences decreased with elevation and the occurrence based rarefaction curves for each elevation did show decreases in slope but no clear asymptote (Fig 3A and 3B). Canopy cover, litter volume and understory height diversity showed weak patterns of increase with elevation while temperature showed a strong decrease (Fig 4A–4D).

### Spatial patterns in species richness

Rarefied species richness for transects across the elevation gradient had strong spatial auto-correlation. However, after accounting for the trend across elevation, the strength of auto-correlation was much lower with lower standard deviates (Table 2). AICc values of regression models with effect of climatic gradient (models 1 and 2, Table 3) were much lower compared to models with habitat variables as the only predictor (Table 3). Temperature was an important predictor variable in regression analysis using other approaches as well (Table 1–4 in S1 Text).

**Table 2:** Moran's I value for observed species richness, Chao2 estimates at each transect and residuals of regression with elevation.

Variable	Moran's I	Standard deviate	p-value
Species richness	0.76	6.75	<0.01
Residuals—species richness	0.24	2.2	0.01

**Table 3:** Results of mixed effects regression models for rarefied species richness with random intercept at each elevation. (MAT = Mean Annual Temperature,).

No.	Variable	Estimate	Std. Error	Random effect	AICc	Deviance
1	Intercept	2.49	0.07	0.01	196.26	189.5
	MAT	0.61	0.08			
2	Intercept	2.48	0.06	0.009	199.68	187.7
	MAT	0.59	0.07			
	Volume of leaf litter	0.07	0.05			
	Understory complexity	0.01	0.05			
3	Intercept	2.47	0.23	0.48	218.34	209.04
	Volume of leaf litter	0.04	0.06			
	Understory complexity	-0.01	0.06			

### Effect of geometric constraints on species richness

The observed number of discontinuous occurrences were much lower than the distribution predicted by the null model (observed relative increment in matrix fill = 0.3, standardised effect size = -6.08, value at 0.025^th^ quantile of the null distribution = 0.46). The model without range cohesion but constrained by temperature (model1) explained only 46% of the variation in species richness (Table 4). This means that species occur at adjacent elevations more often than expected by chance, and limits of temperature on species distribution cannot explain contiguity of ranges. Therefore, we interpolated species occurrences between minimum and maximum for the rest of the analysis.

**Table 4:** Simulation models for species richness of elevation bands. The R^2^ values are calculated as (1 - (deviance of candidate model / deviance of model 1). Model 1 is range scatter model weighted by temperature. Negative R^2^ values indicate that candidate model is not better than the null model.

Models	Description	R2
Model 5	Model4 with upper domain boundary truncated at 2400m	0.78
Model 4	Model2 but midpoint adjustment only at low elevations	0.50
Model 3	Model2 with midpoint adjustment at both boundaries	0.19
Model 2	Hard boundaries, ranges contiguous, temperature weighted	-0.21

The percentage of variation in total species richness explained by 'model3'—combined effect of geometric constraints and temperature—is less than the null model (Table 4). The other two models explained species richness better, with reduced geometric constraints at low elevations but not at high elevations. The best model ('model5') included temperature as a constraining variable and did not have a hard boundary at the lowest observed elevation (Table 4). These results show that species ranges are not limited by geometric constraints at lower elevations, and that distribution of species is limited by temperature (Table 4).

## Discussion

### Decrease in ant species richness with elevation

This study provides the first quantitative analysis of elevational gradients in the diversity of ants from the Eastern Himalaya, a global biodiversity hotspot. We found a linear decrease in observed species richness with elevation without any mid-elevation peak within the observed elevation range. A number of studies on ants from different biogeographic regions such as the Indomalaya [27,54], Nearctic [35], Neotropical [29], Palearctic [55] and Australian [30] regions also report decrease in species richness with elevation, while there are reports of unimodal patterns as well [31,56,57]. Global analysis of interpolated richness suggests that a ‘mid-elevation peak' is the most common pattern but lack of comparable sampling methods and elevational extents limits testing most of the available data [12]. In this study, interpolation did change the pattern from decreasing to a low elevation plateau (sensu [12]; Fig 2 in S1 Text).

Unimodal relationship between species richness and elevation is the most commonly reported pattern elevational richness pattern in the Himalaya [13,58,59,60]. Elevations at which maximum species richness is reported are different among taxa and plant life forms, with peaks for endemic richness at higher elevations than total richness [9,23,61–63]. Ant species richness peaked at 1000m in Western Himalaya, and 2000m site had greater species richness compared to sites at 500m [56]. In comparison, we report species richness at 600m that is three times greater than at 2200m. Therefore, considering the slopes of elevational diversity relation, and the species accumulation curves, a mid- elevation peak is highly unlikely within the spatial scales studied here. Patterns in ant species richness may be unimodal at larger spatial scales due to ecotone between species with tropical and temperate affinities or due to greater proportion of endemic species at mid elevations. These mechanisms could be studied in future with additional inventory effort.

### Edge effects and temperature predict species richness

Geometric constraints may influence species richness patterns across elevations because of the obvious boundaries of sea and summit for terrestrial animals. Many studies from the Himalayan region find little support for geometric constraints or mid-domain hypothesis, and instead, climatic variables appear to predict the patterns better [6,8,23]. The most commonly used geometric constraints model is random placement of ranges within a bounded domain. However, primary data rarely represent spatial extents that will completely cover species ranges. Therefore, at least some species ranges are likely to cross domain boundaries, hence, niche truncation [40] should serve as better null model for most empirical data. The gradient we sampled here is also a truncated subset of the entire elevational range and is continuous with the vast plains of Assam (Fig 1A and 1B). The drastic change in relationship between climate and geographic distance that takes place with the transition from mountains to plains can result in a soft domain boundary at low elevations [39]. Species adapted to conditions on the plains may extend their ranges towards higher elevations or species adapted to intermediate elevations may extend towards the plains. Therefore, limits on species richness due to geometric constraints may not apply at lower elevations.

Among the models we analysed, hard boundaries at both low and high elevations do not explain the observed patterns. The model with the smallest positive R^2^ value was 'Model3' which represents soft boundaries at both ends of the gradient, and limits due to trends in climatic conditions. Models that include asymmetric geometric constraints, with a soft boundary at lower elevations, and a hard boundary at upper elevations increase the model fit further (Table 4). Given that the gradient we sampled is a truncated subset of the entire elevational range, species ranges should be equally likely to overlap either of the domain boundaries. However, the best model in the analysis includes a hard boundary at high elevations, which does not allow any species to extend beyond the domain (Table 4, model 5, R^2^ = 0.72). This is possible if constraints of the climatic variable reduce density of range midpoints at the upper end of geometric constraints so that distributions mimic a hard boundary. The difference between R^2^ values of model2 and model3 highlights this point. This suggests that the magnitude of constraints from the climatic gradients alone is not sufficient to explain the greater overlap of ranges at low elevations but conditions of domain boundaries that reflect nature of landscape are equally important.

All the models together show that species richness of ant communities in EWS is influenced by a combination of climate across the elevational gradient and a soft boundary at lower elevations.

## Temperature, elevation, and species richness

A number of studies across latitude [64,65], elevation [35,66] and other spatial gradients [67] find variables related to temperature to be the best predictor of species richness. Many studies across elevation gradients including global analyses point towards the combined effect of temperature and moisture availability [12]. In this study, precipitation decreased linearly from low to high elevations according to the 'WorldClim' data. Due to the high correlation between temperature and precipitation, it is difficult to separate the effects of the two. However, ant communities in EWS are more likely to be limited by a gradient in temperature, as the average levels of precipitation in the region are high, so water availability may not be limiting. Additionally, actual water availability may be higher towards high elevations due to greater condensation of moisture. Therefore, the decrease in species richness with elevation is very likely caused by decrease in temperature.

While the correlation between temperature and ant species richness is widely documented in a number of studies across elevational gradients, there can be many explanations for this relationship. Here, it is important to dismiss any spurious effects of temperature such as limits on foraging activity and therefore observed species richness. As ants are thermophilic animals, the abundance of foraging workers and foraging time available for ant colonies are most likely limited by ambient temperature [68]. This can cause differences in species richness through sampling biases without any change in actual species richness. Differences in species richness due to such effects should not produce any difference in accumulation functions or rarefied species richness at different elevations [69]. However, this is not the case in EWS. Accumulation curves (Fig 3B) for the lowest elevation (600m) are steeper and higher than other sites. Further, there are distinct differences between 1000m and 1200m, and again between 1800m and 2400m. Therefore, the differences in species richness are unlikely to be entirely due to sampling artefacts.

An alternate ecological mechanism for effect of temperature on species richness is through limits on species composition, where only a small number of species are adapted to colder conditions at higher elevations. This mechanism predicts that variation in climate and particularly variables relating to temperature should best explain variation in species composition, while geographic distance should have minimal effect [70]. Immigration from tropical evergreen source areas along with niche conservatism can lead to a greater number of species adapted to warmer low elevation habitats. Ant species colonizing the Eastern Himalaya from tropical Southeast Asia may retain affinity for tropical conditions and contribute to the negative relationship of species richness with elevation. Molecular evidence suggests that the Himalayan fauna is assembled from immigration and radiation rather than in-situ speciation [71]. Tropical conservatism along with selective colonization and extinction is thought to drive species richness gradients at much larger scales in the Himalaya [72]. Intercepts of the elevational diversity pattern should be higher and slopes should become steeper (more negative) across longitudes from west to east, closer to the centre of diversity where most species have an affinity to tropical conditions. However, testing this hypothesis will require data at much larger scales.

Other mechanisms that can potentially drive the relationship between species richness and temperature include the metabolic theory and energy limitation hypothesis [68,73]. These mechanisms provide general explanations that depend on rates of species diversification and extinction. Here, effects on species richness should be consistent across several elevation gradients, and so the intercept and slope should not have any longitudinal pattern. Further studies at larger spatial scales can shed light on relative importance of these mechanisms.

## Conclusion

Species richness of ant communities in Eaglenest wildlife sanctuary is driven by climatic gradients across elevations and the geographic setting of the Eastern Himalayan mountain range, particularly its continuity with the plains. We arrive at this conclusion by altering geometric constraints at either ends of the gradient independently. This suggests that the pattern in ant species richness in EWS is not due to local effects such as habitat conditions or disturbance but results from general ecological processes.

## Supporting information

S1 Text Methods and results. Explanation for geometric constraints models, interpolating species occurrences, and additional results for geometric constraints models and generalized linear models.
(DOC)

S1 Table Regression data. Species richness and predictor variables used in the regression analysis. The table shows data for observed, rarefied and interpolated species richness along with predictor variables measured at transect and elevation band levels. 'Understory Complexity' and 'Volume of Leaf Litter' were estimated at each transect, and 'Mean Annual Temperature' was taken from 'WorldClim' for each elevation band.
(CSV)

S2 Table Interpolated elevational extents. Data on elevational extents of species used in the geometric constraints analysis. The table shows data on interpolated range extents of species represented by lowest (min) and the highest (max) elevations. 'range' represents the elevational extents, 'mid' the mid-point of the elevational extents, and 'num_bands' the number of elevation bands at which each species is recorded, after interpolation.
(CSV)
