# Supplementary material for: Spatial and climatic variables independently drive elevational gradients in ant species richness in the Eastern Himalaya
Source: PLoS One. 2020 Jan 15;15(1):e0227628. doi: 10.1371/journal.pone.0227628 (PMC6961925; doi:10.1371/journal.pone.0227628)
Supplement: S1 Text — Explanation for geometric constraints models, interpolating species occurrences, and additional results for geometric constraints models and generalized linear models. (DOC) [file pone.0227628.s001.doc]

**Details of explanatory variables used in the regression analysis**

Climate variables: We used climatic variables obtained from 'WorldClim' [46] at 30 arc second resolution as predictors at the level of elevation bands. Because of high correlation among different climatic variables in the WorldClim data, we only used 'Mean Annual Temperature' (Bioclim variable1) to represent the variation in climate across elevation. We calculated pixel values at the four transects and averaged them to get a single value for the grouping variable of elevation. The coarse grain size of the 'WorldClim' data may cause problems relating to data independence when transects from different elevation bands fall in same pixel. In two cases where transects at different elevations were located within the same pixel, we excluded these transects while calculating the mean for the elevation bands.

Volume of Leaf-litter: As measure of complexity of habitat, we estimated volume of leaf litter at each transect. Most ground dwelling ants forage in leaf litter, and its volume should be a good proxy of availability as well as complexity of local habitat for ants. Volume of leaf litter collected for Winklers was estimated using a bucket and approximating its shape to the frustum of a cone.

Complexity of understory vegetation: Understory vegetation may also provide additional habitat for genera that are arboreal foragers such as species of genera *Camponotus* or *Polyrhachis*. The number of stems of saplings or shrubs of different heights in a 1m^2^ quadrat was counted for eight height classes between 1cm to 160cm, at 20cm intervals. Therefore, each quadrat had eight values of number of stems or abundance of vegetation at different heights. Shannon diversity was used to summarize height data within a quadrat. Ten such quadrats were sampled in each 100m transect and averaged.

Temperature: Soil temperature was estimated by inserting a mercury thermometer 10cm below the ground surface at 10 m intervals on the same transect used for trapping ants, and averaged for the transect.

Canopy cover and Tree density: We also estimated canopy cover and tree density at the time of sampling. Canopy cover can limit the amount of sunlight reaching the leaf litter layer and modify local habitat conditions. However, we did not include these in regression analysis as neither variable showed much variation across elevation

**Explanation for the Geometric constraints model**

The following schematic shows possible range locations available for sampling and corresponding adjustment to place the ranges within observed elevational extents. The triangles represent geometric constraints under two different assumptions. The triangle with solid lines represents observed elevational extents and its geometric constraints. The triangle with broken lines shows geometric constraints after assuming that species ranges may extend beyond the lowest and highest elevations sampled. Vertical dotted lines show all possible midpoint range size combinations without considering any geometric constraints. Horizontal lines show examples of possible range locations under each model and arrows indicate shifting of midpoints after random sampling to nearest geometrically possible location. Shifting the midpoints ensures proportion of gradient occupied by species is same as observed data, but it changes the probability of species occurring at domain boundaries.


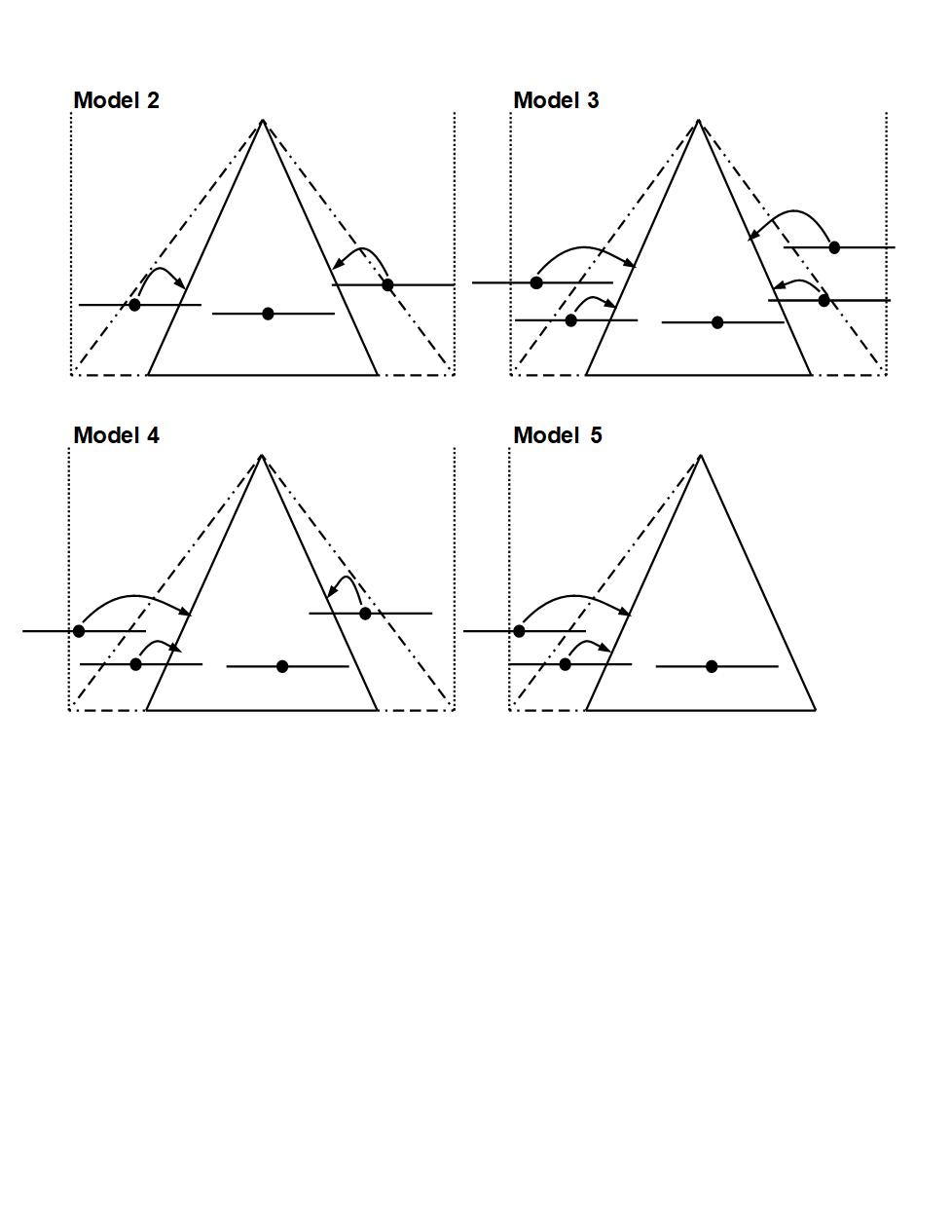
Supplimentary Figure 1. Schematic representation of the geometric constraints models.

**Details of the geometric constraint models used in the analysis**

Model1 – Hard boundaries, ranges not contiguous, temperature weighted – Occurrences across elevation s of each species are randomly assigned to elevations so that the species distribution is not necessarily contiguous. The probability of selecting each elevation is weighted by temperature (Range Scatter + temperature). This model is used as a null model for comparing other models.

Model2 – Model1 with contiguous ranges – Species are entirely confined within the minimum and maximum elevation of the study i.e. the domain extends beyond the sampling range; distributions of each species are strictly contiguous and choice of range location is weighted by temperature.

Model3 – Model2 with midpoint adjustment at both boundaries – Midpoints not possible under geometric constraints on either side of the gradient are shifted to the nearest possible midpoint.

Model4 – Model2 but midpoint adjustment only at low elevations – Same as 'model3' but geometrically impossible combinations towards high elevations cannot be sampled while midpoint adjustment is done at low elevation. This model represents soft boundaries at low elevations and hard boundaries at high elevations.

Model5 - Model4 with upper domain boundary truncated at 2400m – The model is same as 'model4' but the upper boundary of the domain is truncated at 2400m which is the observed upper limit of the study.

Model 1 is the only range scatter model, and includes effects of temperature. We did not include a completely random range scatter model, as there is little biological evidence for such distributions. Goodness of fit for competing geometric constraints models can be compared using the R^2^ values [18,58].

**Interpolated vs. observed ranges**

We compared the observed species occurrences with predictions of 'model1' (range scatter + temperature) to decide if species occurrences should be treated as contiguous (at adjacent elevations). Species occurrences may be contiguous if the species are limited by an environmental variable that shows a trend across elevation. Therefore, we checked if the trend in temperature is sufficient to account for contiguity of ranges. We calculated the discontinuous occurrences as the number of times a species was not recorded between its minimum and maximum elevations. We then produced 100 randomized matrices using 'model1' and calculated the same measure for each matrix. We compared the number of discontinuous occurrences in the observed matrix with those predicted by 'model1'.

If accounting for trend in temperature alone is not sufficient to explain contiguity in species occurrences, then the observed discontinuous occurrences should be less than predicted by the model. We chose interpolated species ranges following the results of this analysis. We used package 'rangemodelR v1.0.4' ([https://CRAN.R-project.org/package=rangemodelR](https://cran.r-project.org/package=rangemodelR) ) in 'R v3.4' [40] for running geometric constraints models.

**Results**


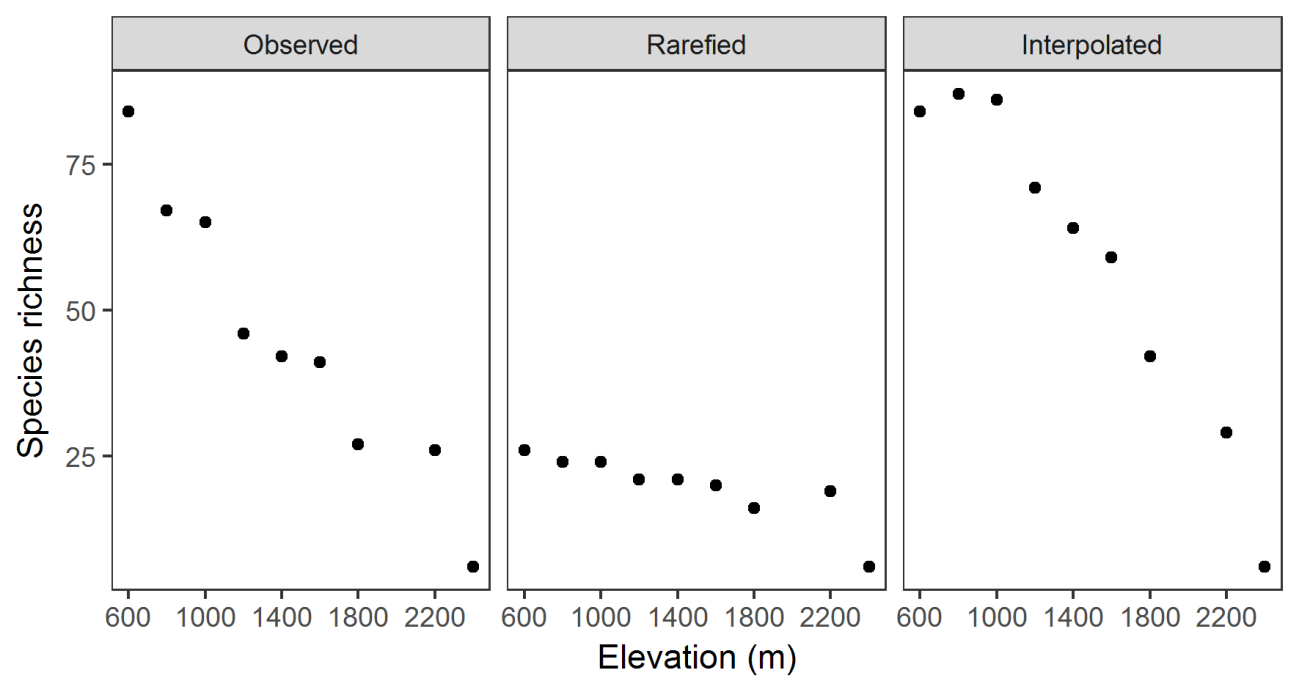


**Supplementary Figure 2.** Observed, interpolated, and rarefied species richness of elevation zones across elevation.

**Supplementary Table 1**. Generalized linear models with poison errors for observed species richness at transects (N = 36).

| Model | Variables | Estimate | Std. Error | AIC | Deviance |
| --- | --- | --- | --- | --- | --- |
| 1 | Intercept  Temperature  Understory complexity  Leaf litter volume | 2.9  0.60  0.09  0.03 | 0.04  0.04  0.04  0.04 | 220.5 | 42.99 |
| 2 | Intercept  Temperature  Understory complexity | 2.9  0.5  0.09 | 0.04  0.04  0.04 | 219.2 | 43.71 |
| 3 | Intercept  Temperature | 2.9  0.57 | 0.04  0.03 | 222.2 | 48.65 |

**Supplementary table 2.** Generalized linear models with poison errors for species richness rarefied among transects within an elevation bin to account for difference in sample size (N = 36).

| Model | Variables | Estimate | Std. Error | AIC | Deviance |
| --- | --- | --- | --- | --- | --- |
| 1 | Intercept  Temperature  Understory complexity  Leaf litter volume | 2.5  0.5  0.07  0.09 | 0.05  0.05  0.05  0.04 | 197.5 | 35.39 |
| 2 | Intercept  Temperature  Leaf litter volume | 2.5  0.5  0.09 | 0.05  0.04  0.04 | 197.7 | 37.52 |
| 3 | Intercept  Temperature | 2.5  0.5 | 0.04  0.04 | 199.63 | 41.44 |

**Supplementary table 3.** Generalized linear models with poison errors for interpolated species richness of elevation bins. These models are single predictor models to avoid over fitting (N = 9).

| Model | Variables | Estimate | Std. Error | AIC | Deviance |
| --- | --- | --- | --- | --- | --- |
| 1 | Intercept  Temperature | 3.9  0.4 | 0.04  0.05 | 88.1 | 32.76 |
| 2 | Intercept  Leaf litter volume | 4.06  0.15 | 0.04  0.04 | 181.69 | 126.35 |
| 3 | Intercept  Understory complexity | 4.06  0.08 | 0.04  0.04 | 189.11 | 133.77 |

**Supplementary table 4.** Generalized linear models with poison errors for rarefied species richness of elevation bins. These models are single predictor models to avoid over fitting (N = 9).

| Model | Variables | Estimate | Std. Error | AIC | Deviance |
| --- | --- | --- | --- | --- | --- |
| 1 | Intercept  Temperature | 2.9  0.27 | 0.07  0.08 | 53.37 | 6.5 |
| 2 | Intercept  Leaf litter volume | 2.9  -0.008 | 0.07  0.07 | 64.48 | 17.64 |
| 3 | Intercept  Understory complexity | 2.9  0.06 | 0.07  0.07 | 63.86 | 17.02 |

**Supplementary table 5.** Simulation models for species richness of elevation zones. Here classical R^2^ values are reported for reference.

| **Models** | **Description** | **R2** |
| --- | --- | --- |
| Model 5 | model 4 + extended domain and midpoint adjustment only at low elevation | 0.86 |
| Model 4 | model 2 + extended domain at both boundaries but midpoint adjustment only at low elevation | 0.68 |
| Model 3 | model 2 + extended domain and midpoint adjustment at both boundaries | 0.48 |
| Model 2 | Model 1 with contiguous ranges + extended domain at both boundaries | 0.22 |

**Explanation of column headers for response variables in ‘S1’**

‘observed_species_richness_transect’: Observed species richness at each transect, using all the methods.

‘rarefied_species_richness_transect’: Rarefied species richness for a common sample size of 10 Winklers at each transect

‘observed_species_richness_elevation’: Obseved species richness at each elevation band, after pooling all the four replicates together

‘interpolated_species_richness_elevation’: Species richness of each elevation band after interpolating species occurrences, between the minimum and maximum elevation at which every species is recorded.

‘rarefied_species_richness_elevation’: Rarefied species richness at elevation bands, for 40 occurrences which is the smallest number of occurrences recorded in this study (at 2400m).
